# From bedside to bench and back again: translational studies of mechanical unloading of the left ventricle to promote recovery after acute myocardial infarction

**DOI:** 10.12688/f1000research.14597.1

**Published:** 2018-11-27

**Authors:** Navin K. Kapur, Shiva Annamalai, Lara Reyelt, Samuel J. Karmiy, Allen A. Razavi, Sina Foroutanjazi, Aditya Chennojwala, Kiyotake Ishikawa

**Affiliations:** 1 The Molecular Cardiology Research Institute, Tufts Medical Center and Tufts University School of Medicine, Boston, Massachusetts, USA; 2 The Acute Mechanical Circulatory Support Working Group, Tufts Medical Center and Tufts University School of Medicine, Boston, Massachusetts, USA; 3 The Cardiovascular Center, Tufts Medical Center and Tufts University School of Medicine, Boston, Massachusetts, USA; 4 Cardiovascular Research Center, Mount Sinai School of Medicine, New York, New York, USA

**Keywords:** Unloading, acute myocardial infarction, mechanical circulatory support, preclinical models

## Abstract

Heart failure is a major cause of global morbidity and mortality. Acute myocardial infarction (AMI) is a primary cause of heart failure due in large part to residual myocardial damage despite timely reperfusion therapy. Since the 1970’s, multiple preclinical laboratories have tested whether reducing myocardial oxygen demand with a mechanical support pump can reduce infarct size in AMI. In the past decade, this hypothesis has been studied using contemporary circulatory support pumps. We will review the most recent series of preclinical studies in the field which led to the recently completed Door to Unload ST-segment Elevation Myocardial Infarction (DTU-STEMI) safety and feasibility pilot trial.

## Heart attacks lead to heart failure

**Figure 1.**
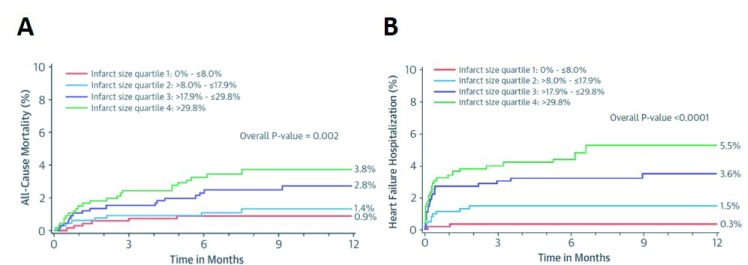
Infarct size as a correlate of heart failure onset. All-cause mortality ( **A**) and heart failure hospitalization ( **B**), showing a strong association between infarct size and adverse outcomes during 1-year follow-up.

Acute myocardial infarction (AMI) is a leading cause of heart failure (HF) with an annual incidence of 805,000 in the US alone. Since 1960, mortality due to AMI has decreased because of improved medical therapy, the advent of coronary angioplasty and stenting, and community-based team efforts in establishing a system of care for AMI ^1^. Parallel to improved survival after AMI, the incidence of heart failure has grown exponentially over the same time period ^2^. By 2030, more than 8 million individuals in the US are projected to have heart failure (1 in every 33 individuals) with an estimated cost of $70 billion in health-care expenditure ^3^. One explanation for the growing population of patients with heart failure is that the magnitude of myocardial damage due to a heart attack correlates directly with an increase in subsequent heart failure ( Figure 1).

In 1971, Maroko, Braunwald, and their team conducted a series of preclinical studies exploring novel approaches to limit myocardial damage due to an acute coronary occlusion ^4^. They reported that “measures designed for reduction of myocardial oxygen demands and improvement of coronary perfusion, when effected promptly after a patient has been brought to a hospital, might potentially reduce the ultimate size of the infarction” ^4^. This landmark study introduced the concept of targeting myocardial oxygen supply or demand to limit myocardial damage in AMI. Soon after this report, in 1976, a young physician-innovator, Andreas Gruentzig, reported his preclinical studies ****testing the concept of restoring myocardial oxygen supply by inflating a balloon in an occluded or stenotic coronary vessel ^5^. Within 1 year, the first coronary angioplasty was performed ^5, 6^, and primary percutaneous coronary intervention has since evolved to be the preferred primary reperfusion approach for AMI therapy to reduce myocardial infarct size ^7^.

Four decades later, primary reperfusion using balloon angioplasty as a widely accepted metric of clinical quality is recommended within 90 minutes from first medical contact in patients presenting with ST-segment elevation myocardial infarction (STEMI) ^8^. This approach has significantly reduced mortality associated with AMI; however, despite primary reperfusion, median infarct size remains between 18% and 40% ^7, 9, 10^. An analysis of over 7,000 patients with AMI identified that about 75% of patients develop heart failure within 5 years of their initial infarct ^11^. Another recent analysis, of over 2,600 patients with AMI treated with contemporary therapy, showed that every 5% increase in myocardial infarct size is associated with a 20% increase in 1-year hospitalization for heart failure and 1-year mortality ^7^. For these reasons, reducing myocardial damage during a heart attack remains a major unmet clinical need.

## Comparative studies on mechanical circulatory support devices

**Figure 2.**
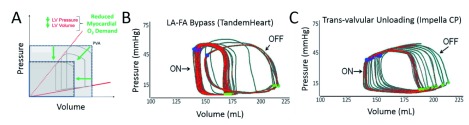
Left ventricular (LV) unloading and pressure volume (PV) area figure. ( **A**) Relationship between pressure and volume and the myocardial oxygen demand. ( **B**) PV loop on and off left atrial to femoral artery (LA-FA) bypass (TandemHeart). ( **C**) PV loop on and off trans-valvular unloading (Impella CP).

Prior attempts to limit myocardial damage during AMI have focused on vascular conditioning or pharmacologic approaches; despite promising outcomes of preclinical and some pilot early clinical studies, these approaches failed to show clear clinical benefit ^12, 13^. One of many potential barriers contributing to these unsuccessful strategies may be current practice focusing on rapid coronary reperfusion, which leaves insufficient time for these strategies to have any beneficial impact prior to reperfusion. Over the past two decades, advances in bioengineering have developed percutaneously delivered acute circulatory support rotary flow pumps, including the TandemHeart (TH) (LivaNova, London, UK) and Impella (Abiomed Inc., Danvers, MA, USA) devices ^14^. The TH is a percutaneously deployed left atrial-to-femoral artery bypass pump that draws blood from the left atrium into an extracorporeal centrifugal flow pump, which delivers flow back into a femoral artery. The Impella CP is a percutaneously deployed micro-axial flow catheter that is delivered via a femoral or axillary artery into the left ventricle (LV) and displaces blood from the LV to the aorta. Both devices reduce myocardial oxygen consumption ( Figure 2) ^15, 16^. Several recent preclinical reports compared the TH and Impella CP devices and identified that both devices reduce LV stroke work (LVSW) and myocardial oxygen demand ^17, 18^. The TH device reduced LVSW by decreasing LV volume, whereas the Impella CP reduced both LV pressure and volume, known as LV unloading. Since then, multiple preclinical studies have begun to explore the impact of these devices on myocardial function and recovery after AMI ^19– 28^.

## Preclinical studies on left ventricular unloading

**Figure 3.**
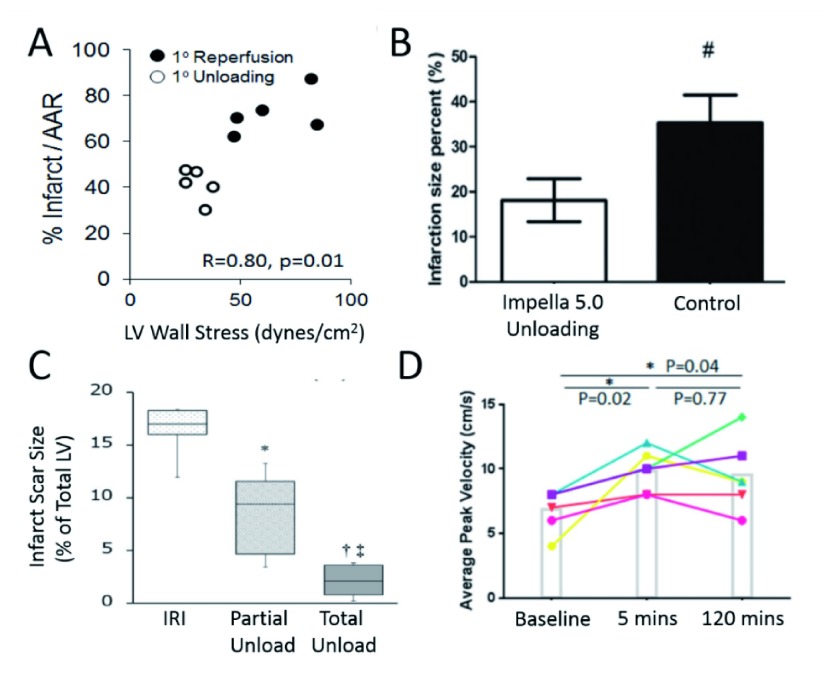
Association between infarct percentage and left ventricular (LV) wall stress ( **A**), infarct percentage after Impella 5.0 unloading (# *p*<0.05 versus Impella 5.0 unloading) ( **B**), infarct scar size and unloading (* *p*<0.05 versuss IRI; † *p*<0.001 versus IRI; ‡ *p*<0.05 versus partial unload) ( **C**), and average peak velocity over time ( **D**) AAR, area-at-risk; IRI, ischemia reperfusion injury.

Multiple preclinical studies have explored the impact of LV unloading using various pumps, including the intra-aortic balloon pump, TH, and Impella, on myocardial infarct size during AMI. A common theme among early preclinical studies is that unloading before reperfusion, not after, is required to reduce infarct size ^23^. These studies further identified multiple mechanisms by which ventricular unloading limits myocardial damage, including improvement in myocardial calcium handling, protection against mitochondrial destruction, and improved myocardial perfusion ^24^. Over the past 3 years, several studies have provided key mechanistic insights into the potential for LV unloading in AMI as a therapeutic strategy to improve outcomes after AMI ( Figure 3).

In 2015, using a porcine model of AMI, we tested the hypothesis that compared with immediate reperfusion (primary reperfusion), first unloading the LV and delaying reperfusion for 60 minutes (primary unloading) would reduce myocardial damage ^25^. In this study, the left anterior descending (LAD) artery was occluded for 90 minutes followed by immediate reperfusion for 120 minutes in the primary reperfusion group. In the primary unloading group, after 90 minutes of LAD occlusion, an Impella CP device was activated and the LAD was left occluded for an additional 60 minutes (total LAD occlusion time of 150 minutes) and then reperfused for 120 minutes. After reperfusion, compared with primary reperfusion, the primary unloading group had significantly reduced LV mean wall stress and peak wall stress. Compared with primary reperfusion, primary unloading reduced LV infarct size by 43% and the magnitude of infarct reduction correlated directly with the magnitude of wall stress reduction. We further identified for the first time that compared with primary reperfusion, primary unloading increased protein levels and activity of the cardioprotective cytokine stromal derived factor 1 alpha (SDF1a). These findings suggested for the first time that first unloading the LV and delaying coronary reperfusion may significantly reduce myocardial infarct size compared with primary reperfusion alone. However, several questions remained unanswered: (1) what were the long-term effects of primary unloading on LV scar size and LV function, (2) how important was the delay to reperfusion and was 60 minutes required, and (3) was SDF1a an innocent bystander or did it play a functional role in the cardioprotective effects of primary unloading ^26^?

The answer to the first question began to develop 1 year later when Sun *et al*. performed a similar study in a porcine model of AMI and employed an Impella 5.0 left direct (LD) trans-valvular pump ^22^. In this study, the left circumflex was surgically ligated in 12 pigs for 120 minutes followed by 120 minutes of reperfusion. The unloaded group received Impella LD support beginning 90 minutes after the start of ischemia (30 minutes before reperfusion). Compared with AMI controls (without Impella LD support), the unloaded group had significantly lower LV pressures, higher mean arterial pressure and cardiac output, and reduced wall stress. After 120 minutes of reperfusion, animals were kept alive for long-term follow-up. One month after AMI, the unloaded group showed reduced infarct size compared with controls. The authors concluded that early unloading using a surgically implanted Impella LD pump in AMI may improve long-term prognosis by reducing LV scar size after AMI. However, the translation of this study to clinical practice is limited by the need for surgical implantation of an Impella 5.0 pump.

In 2018, Saku and Sunagawa explored the effect of different degrees of LV unloading by using an Impella CP device in a canine model of AMI ^27^. In this model, the LAD was occluded for 180 minutes and then reperfused for 60 minutes. In the partially unloaded group, the Impella CP flow was increased until arterial pulse pressure was reduced by half. In the totally unloading group, the Impella CP was activated until the arterial waveform was non-pulsatile. In contrast to controls (AMI without pump support), unloaded animals received activation of the Impella CP 60 minutes after the onset of ischemia (120 minutes before reperfusion) and continued until 60 minutes after reperfusion. In this survival model, all animals were kept alive and monitored for 4 weeks. Partial and full Impella CP support, when compared with controls, during the acute phase of AMI, reduced infarct size by 48% and 87%, respectively. These findings supported the findings by Sun *et al*. and further suggested that early activation of a percutaneously delivered Impella CP device could reduce late-term LV scar size after AMI. However, translation of this study was limited by the need to initiate Impella support 60 minutes after the onset of ischemia.

In the same year as Saku and Sunagawa, Watanabe and Ishikawa reported that mechanical LV unloading with an Impella CP pump increased microvascular perfusion and decreased LV end-diastolic wall stress (EDWS) after AMI when compared with pharmacological unloading ^19^. In this study, AMI was induced in 11 Yorkshire swine and 2 weeks later the animals were divided into two groups: mechanical unloading by Impella CP (n=6) and pharmacological unloading by intravenous sodium nitroprusside (SNP) infusion (n=4). Both groups showed significant decreases in LV wall stress, although this was accompanied by severe hypotension (<60 mm Hg) in two of the four pigs in the pharmacological group. Epicardial coronary flow in the LAD and left circumflex arteries was increased in the Impella group while remaining at similar levels in pigs treated with SNP. Microvascular perfusion to the infarcted area was increased twofold in the Impella group, while no significant difference was seen in the SNP group. Additionally, microvascular perfusion to the infarct area was shown to inversely correlate with LV EDWS in all animals studied (r ^2^ = 0.43), suggesting the key role of wall stress in regulating tissue perfusion in the infarcted heart. Mechanical LV unloading with left ventricular assist device (LVAD) during subacute MI seemed to be superior to pharmacological unloading by decreasing LV EDWS and increasing coronary flow and microvascular perfusion to the infarcted zone without causing hypotension. This study provided key mechanistic insight by suggesting that in addition to reducing LV wall stress and myocardial oxygen consumption, LV unloading may improve microcirculatory blood flow after AMI, thereby promoting myocardial recovery.

**Figure 4.**
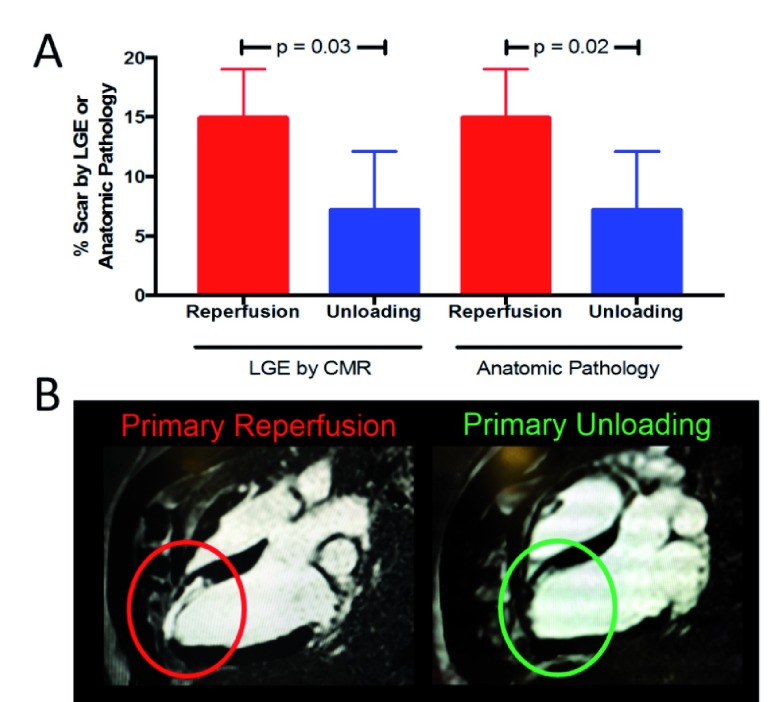
Late gadolinium enhancement (LGE) and anatomical pathology evidence ( **A**) as well as magnetic resonance imaging evidence ( **B**) of reduced scar percentage due to unloading CMR, cardiovascular magnetic resonance.

Soon after these two reports in 2018, we reported findings from a pig study designed to explore the kinetics of delayed reperfusion and provide more mechanistic insight into the cardioprotective role of primary unloading ^28^. In this study, we first determined that activation of an Impella CP for 30 minutes before reperfusion was necessary and sufficient to reduce infarct size by over 40%. Activation of the Impella CP for 15 minutes before reperfusion or immediately after reperfusion did not significantly reduce infarct size. Next, using whole transcriptome analysis of the infarct zone, we found that compared with primary reperfusion, primary unloading increased the expression of genes associated with cellular respiration and mitochondrial integrity. To further explore the functional role of SDF1a, we first identified that levels of proteases known to degrade SDF1a, such as matrix metalloproteinase-2 (MMP-2), MMP-9, and dipeptidyl peptidase-4 (DPP-4), were decreased after primary unloading but not after primary reperfusion. Moreover, inhibiting SDF1a activity attenuated the reduction in infarct size associated with primary unloading. Finally, we performed a survival study, whereby swine were assigned to either primary reperfusion or primary unloading and monitored for 28 days after AMI. We reported that compared with primary reperfusion, primary unloading for 30 minutes before reperfusion reduced LV scar size, improved cardiac function, and limited the expression of biomarkers associated with heart failure and maladaptive cardiac remodeling ( Figure 4). These findings identified for the first time that 30 minutes of LV unloading prior to reperfusion was sufficient to achieve a reduction in infarct size and provide evidence that LV unloading activates a cardioprotective signaling program that results in a durable reduction in myocardial damage after AMI.

## Clinical Translation of LV Unloading in AMI

Combined with decades of prior preclinical studies, these five recent studies supported the development of a clinical trial designed to test the safety and feasibility of LV unloading prior to reperfusion using a percutaneously delivered trans-valvular axial flow device. The Door To Unloading With IMPELLA CP System in Acute Myocardial Infarction (DTU) Study (ClinicalTrials.gov Identifier: NCT03000270) is a prospective, multi-center, randomized phase I trial testing whether direct active unloading of the LV with the Impella CP prior to reperfusion is feasible and safe in patients with anterior STEMI. A total of 50 patients presenting with an initial anterior STEMI without cardiogenic shock were randomly assigned to receive Impella CP support followed by either immediate reperfusion (U-IR) or a 30-minute period of LV unloading prior to reperfusion (U-DR). In both arms, the Impella CP was activated prior to reperfusion. All patients completed the U-IR (n=25) or U-DR (n=25) protocols with mean door-to-balloon times of 72 versus 97 minutes, respectively. Rates of major adverse cardiovascular and cerebral events (MACCE) at 30 days after STEMI were low and not different between the U-IR versus U-DR groups (two versus three events, respectively, *p*=0.99). Compared with the U-IR group, delaying reperfusion in the U-DR group did not increase mean infarct size normalized to either area at risk or total LV mass at 3–5 days or 30 days after STEMI, respectively. No patients assigned to the U-DR arm received bailout coronary reperfusion before the protocolized 30-minute delay. An exploratory subgroup analysis identified that among patients with larger anterior STEMIs, as adjudicated by sum ST-segment elevation, unloading and delaying reperfusion had a statistically significant lower infarct size normalized to the area at risk compared to unloading and immediate reperfusion. The DTU-STEMI pilot trial is the first study to report that LV unloading using the Impella CP device with a 30-minute delay before reperfusion is feasible within a relatively short time period in anterior STEMI. The DTU-STEMI pilot trial did not identify prohibitive safety signals that would preclude proceeding to a larger pivotal study of LV unloading before reperfusion. An appropriately powered DTU-STEMI pivotal trial comparing LV unloading before reperfusion to the current standard of care is now in development ^26^.

## Conclusions

Given the exponential growth in patients with heart failure due to ischemic heart disease, there is an inherent need for therapies that limit myocardial damage after AMI. No clinical studies of cardioprotection have been able to successfully extend the benefit of primary reperfusion. Given the studies described above, it may be that the ultimate approach to further reduce myocardial damage is to adhere to the advice first provided by Maroko and Braunwald in 1971, which was to reduce myocardial oxygen demand and then increase coronary perfusion in AMI. Whether primary unloading represents the next step for cardioprotective therapy remains to be proven in a randomized clinical trial, but primary unloading, if successful, may shift the management paradigm for AMI and improve clinical outcomes for millions of patients at risk of developing heart failure after an AMI.
